# Synthesis and Characterization of NiCoPt/CNFs Nanoparticles as an Effective Electrocatalyst for Energy Applications

**DOI:** 10.3390/nano12030492

**Published:** 2022-01-30

**Authors:** Esam E. Abdel-Hady, Mohamed Shaban, Mohamed O. Abdel-Hamed, Ahmed Gamal, Heba Yehia, Ashour M. Ahmed

**Affiliations:** 1 Physics Department, Faculty of Science, Minia University, Minia 61519, Egypt; esamhady@yahoo.com (E.E.A.-H.); mazosman2005@yahoo.com (M.O.A.-H.); hdody393@gmail.com (H.Y.); 2 Academy of Scientific Research and Technology (ASRT) of the Arab Republic of Egypt, Cairo 11516, Egypt; 3 Department of Physics, Faculty of Science, Islamic University in Madinah, Al-Madinah Al-Munawarah 42351, Saudi Arabia; 4 Nanophotonics and Applications (NPA) Lab, Physics Department, Faculty of Science, Beni-Suef University, Beni-Suef 62514, Egypt; a_gamal21@yahoo.com (A.G.); ashour.elshemey@gmail.com (A.M.A.)

**Keywords:** Ni-Co-Pt/CNFs, electrocatalyst, ethanol, methanol, urea, electrooxidation, impedance spectroscopy

## Abstract

In this work, three nanoparticle samples, Ni_4_Co_2_Pt/CNFs, Ni_5_CoPt/CNFs and Ni_6_Pt/CNFs, were designed according to the molar ratio during loading on carbon nanofibers (CNFs) using electrospinning and carbonization at 900 °C for 7 h in an argon atmosphere. The metal loading and carbon ratio were fixed at 20 and 80 wt%, respectively. Various analysis tools were used to investigate the chemical composition, structural, morphological, and electrochemical (EC) properties. For samples with varying Co%, the carbonization process reduces the fiber diameter of the obtained electrospun nanofibers from 200–580 nm to 150–200 nm. The EDX mapping revealed that nickel, platinum, and cobalt were evenly and uniformly incorporated into the carbonized PVANFs. The prepared Ni-Co-Pt/CNFs have a face-centered cubic (FCC) structure with slightly increased crystallite size as the Co% decreased. The electrocatalytic properties of the samples were investigated for ethanol, methanol and urea electrooxidation. Using cyclic voltammetry (CV), chronoamperometry, and electrochemical impedance measurements, the catalytic performance and electrode stability were investigated as a function of electrolyte concentration, scan rate, and reaction time. When Co is added to Ni, the activation energy required for the electrooxidation reaction decreases and the electrode stability increases. In 1.5 M methanol, the Ni_5_CoPt/CNFs electrode showed the lowest onset potential and the highest current density (30.6 A/g). This current density is reduced to 28.2 and 21.2 A/g for 1.5 M ethanol and 0.33 M urea, respectively. The electrooxidation of ethanol, methanol, and urea using our electrocatalysts is a combination of kinetic/diffusion control limiting reactions. This research provided a unique approach to developing an efficient Ni-Co-Pt-based electrooxidation catalyst for ethanol, methanol and urea.

## 1. Introduction

Energy is a crucial factor that affects both economic progress and human survival. Concerns about human health and environmental hazards have grown significantly in recent decades. Many of these problems, such as acid rain, ozone depletion, and global climate change, are related to energy production, transformation, and consumption. Today, energy supplies are running out, prompting a greater desire to find clean renewable alternative fuels. Polymer-electrolyte membrane fuel cells (PEMFCs) are a potential solution for producing clean energy. PEMFCs have received a lot of attention due to their high efficiency, high power density, ease of scale-up, and low operating temperatures [1]. However, the high cost of Pt catalysts, combined with limited resources, has sparked interest in developing free or low-content platinum group metal catalysts for use in PEMFCs [2]. As a result, the following two strategies are being used to reduce Pt usage. The first is to reduce the size of the catalyst particles, resulting in a higher specific surface area. At the nanoscale, size has been found to influence catalytic properties, whereas NPs in the 1–30 nm range exhibit better catalytic efficacy as size decreases [3]. Also, the crystallographic growth plane of the catalyst was found to have an impact on the catalytic property. For example, the reaction rate on Pt {111} was found to be roughly five times that of the {100} plane in the dehydrogenating cyclization process of n-butane [4]. The second strategy is to combine Pt with less expensive elements with moderate catalytic activity, such as Co, Ni, Fe, and others [5,6,7,8,9,10].

Noble metallic nanoparticles (NPs) and carbon-based materials of various sizes, shapes, facets, and compositions are widely used in the catalytic industries because of their distinct properties [11,12,13,14,15,16,17,18,19,20]. For example, Pt is used in automobiles to convert harmful exhaust gases such as CO, NOx, and hydrocarbons into CO_2_, N_2_ and H_2_O. Also, Pt is widely employed as a catalyst in the fuel cell, however, the lethargic oxygen reduction (OR) and its slow kinetics on Pt being claimed to limit the operation of the fuel cells [21,22,23,24,25]. Many groups have recently conducted several studies to improve Pt catalysts’ performance in the negative electrode, including their activity and stability [11,12]. Different cathodic catalysts based on Pt alloys have been developed such as Pt/Fe, Pt/Co, Pt/Ni, and Pt/Cr [26,27,28,29,30,31,32]. These catalysts have been employed in PEMFCs for hydrogen oxidizing at the anode and the oxygen reduction occurs at the cathode due to the low overpotential and strong catalytic activity [26]. Also, Pt is alloyed with 3d transition metal to achieve high activity at 0.9 V_iR-free_ for the oxygen-reducing reaction (ORR) [28,33,34,35,36,37,38]. Compared to pure Pt in PEMFCs, Pt-alloys containing different transition metals, such as Ni, Cr, Co, and others, have shown increased ORR electrocatalytic activities [29,36,37,38,39]. This improvement can be attributed to a variety of factors, including a decrease or reduction in the Pt oxidation state, suppression of Pt oxide formation, formation of new electronic structures with increased Pt 5d orbital vacancies, improvement of O_2_ adsorption due to the decrease in the Pt-Pt interatomic distance, formation of a thin Pt skin on the alloy core surfaces, and modified electronic structure of the highest-ranked Pt atoms [34,36,37,40,41,42,43,44,45,46].

The PtCo catalyst has gained a lot of attention amongst Pt-alloy catalysts because of its high stability and activity in acidic environments [29,47]. Paulus et al. looked at bulk Pt compositions of 50 and 75 percent Pt with Ni and Co alloying components [35,48]. The 25 at. percent Ni and Co catalysts showed a small activity rise of around 1.5 times, while the 50 at. percent Ni and Co catalysts showed a more substantial enhancement of factor 2–3 when compared to pure Pt. For the ORR, Huang et al. found that PtCo alloy nanoparticles have activity and specific activity improvements of 1.3–3.2 and 1.2–2.2, respectively, when compared to pure Pt [49]. Antolini et al. found that PtCr and PtCo are significantly more stable in terms of catalytic activity and stability than PtV, PtNi and PtFe due to their high degree of alloying and particle size [29]. Jayasayee et al. investigated the activity and durability of PtCo, PtNi and PtCu in PEMFC cathodes as a function of alloying elements [38]. Relative to PtNi and Pt electrocatalysts, the performances of PtCo and PtCu electrocatalysts were observed to be the most appealing. The activities of PtCu, PtCo and PtNi alloys in PEMFCs were examined by Mani et al. [39]. Pt-alloys containing Co and Cu were shown to be more active than PtNi. Relative to the commercial Pt/C electrocatalyst, the masses and specific activities of PtCo and PtCu were increased by a ratio of 3–4.

Although these attempts, the commercialization of Pt-based PEMFCs for several applications; including portable electronics, automotive, and stationary electricity; is still required higher durability, lower cost, and higher efficiency than the existing levels. Also, commercial fuel cell lifetime requirements vary greatly depending on the application: 5000 h for vehicles, 20,000 h for buses, and 40,000 h for immobile applications [33]. So, the development of morphologically and structurally managed Pt nanostructures or innovative Pt-based nanocomposites is urgently required to meet the ever-increasing demand in energy and related fields.

In this work, we suggest the design of a particulate nanocomposite from Ni and Co with a limited ratio of Pt (3%) and the use of a low-cost supporting nanofibrous for the mass production of a low-cost nano electrocatalyst toward the design of commercial fuel cells. This work aims to fabricate and characterize a low-cost electrocatalyst utilizing Co and Ni as replacements of the Pt. Here, NiCoPt/CNFs nanoparticles-incorporated carbon nanofibers are designed, characterized, and investigated as electrocatalysts for electrooxidation of ethanol, methanol, and urea. The influence of the Ni/Co ratio on electrocatalyst morphologies, structures, and electrooxidation performance was examined. The electrocatalysts are characterized by X-ray diffraction (XRD), energy dispersive X-ray mapping (EDX), transmission electron microscopy (TEM), and scanning electron microscopy (SEM). The electrocatalytic properties of the samples are studied for electrooxidation of ethanol, methanol, and urea in an alkaline medium. The effects of electrolyte concentration, scan rate, and reaction duration on the performance of the proposed catalysts are investigated. Furthermore, the electrode stability and performance are examined using cyclic voltammetry (CV), chronoamperometry, and electrochemical impedance spectroscopy (EIS) measurements. The Ni-Co-Pt ternary nanoparticles are likely to be useful as a free catalyst in fuel cell applications.

## 2. Experimental Details

### 2.1. Samples Preparation

**Figure 1:**
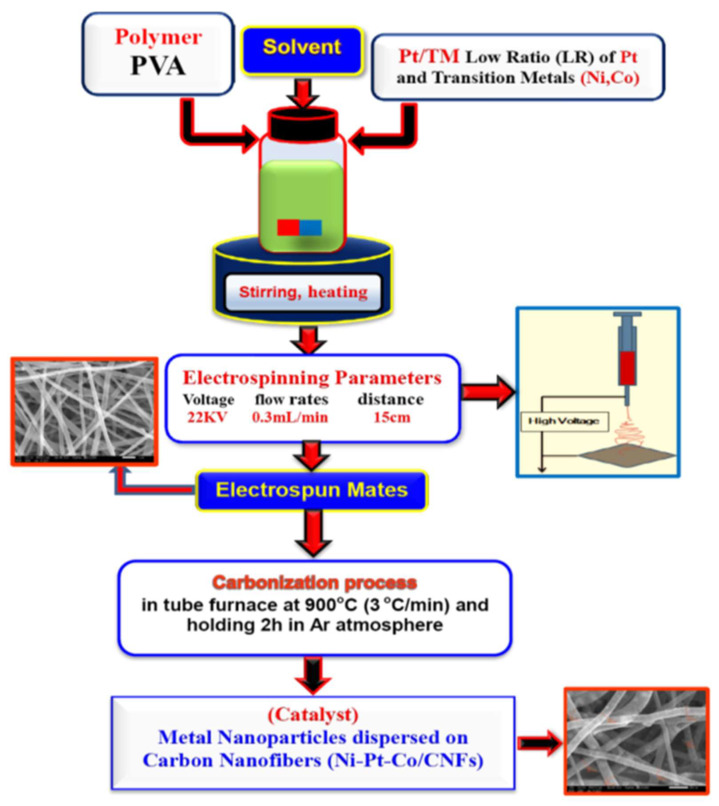
Schematic illustration of the synthetic procedure of Metal embedded in Carbon Nanofibers (MNPs/CNFS).

Figure 1 shows a schematic illustration of the synthetic procedure of metal embedded in carbon nanofibers (MNPs/CNFS). Solutions of PVA (Alpha, Germany) 10 wt% concentration in water containing Nickel (II) acetate tetrahydrate (NiAc, 98% (Alpha, Germany)), Cobalt acetate (CoAc, 98% (Alpha, Germany)), and H_2_PtCl_6_ (Alpha, Germany). The theoretical metal loading and carbon ratio were fixed at 20 and 80 wt%, respectively. The theoretical Ni:Co:Pt molar ratio is 55:30:15, 70:15:15 and 85:15 for Ni_4_Co_2_Pt/CNFs, Ni_5_CoPt/CNFs and Ni_6_Pt/CNFs, respectively. Typically, 1.2896:1.9930 gm NiAc (Ni_11:17wt%_), 0.25–0.5 gm CoAc (Co_3–6wt%_) and 0.1755 gm H_2_PtCl_6_ (Pt_3wt%_) were mixed with PVA solution to make a combination of 80 weight percent polymer and 20 weight percent (Ni/Co/Pt) of various mass ratios. For all samples, total metal loading was kept at 20%, with varying percentages of particular metals as shown in Table 1. The mixture was stirred at room temperature (RT) overnight until it obtained a clear and homogeneous solution. Homogeneous electrospinning solutions were loaded in 20 mL. Positive voltage, working distance, and flow rate were the optimal Electrospinning parameters, which were 22 kV, 15 cm and 0.3 mL/h, respectively. On the surface of a metal plate, nanofibers were accumulated as a fibrous web. The electrospun PVA/Ni(Ac)-Co(Ac)-H_2_PtCl_6_ nanofibers were dried in a vacuum at 80 °C for 24 h before being carbonized in an argon environment at 900 °C for 7 h (heating rate 3 °C/min, holding 2 h).

### 2.2. Samples Characterization

A scanning electron microscope (SEM) (Jeol JSM-I T 200, Tokyo, Japan) equipped with energy dispersive X-rays (EDX) was used to examine the surface morphology of the nanofibers. Using an X-ray diffractometer (202964/Panalytical Empryan, Malvern, UK) and Cu K_α_ (λ = 0.1540 Å) radiation, the structure and crystallinity of the produced catalyst were investigated. The diffraction pattern was investigated over a range from 10° to 100° at a step size of 0.05. An electrochemical analyzer (CHI 660E Series, Austin, TX, USA) was used to measure the electrocatalytic activities of electrodes as anode or cathode for methanol or ethanol electro-oxidation. The electrochemical cell consisted of three electrodes: a reference electrode (Ag/AgCl; KCl solution concentration was 4.0 M), an auxiliary (counter) electrode (Pt wire), and the working electrode (Ag/AgCl) (glassy carbon electrode; GC).

### 2.3. Electrochemical (EC) Measurements

The electrochemical analysis was performed in a typical three-electrode cell utilizing an electrochemical analyzer (CHI 660E Series, Austin, TX, USA). The experimental set-up and all its components were shown in Figure S1 (supplementary data).

The working electrodes were made in the following manner: In a tiny agate mortar, 5 mg of catalyst powder and 50 µL Nafion were disseminated in 0.4 mL Isopropyl alcohol and then crushed into a slurry. To make a homogenous catalyst ink, the mixture was stirred for 12 h. A slurry of around 16 µL was then applied directly to a glassy carbon electrode of 0.07065 cm^2^ surface area. The electrode was then dried for 1 h at RT. Pt wire was used for the counter electrode, while Ag/AgCl was used for the reference electrode. As demonstrated in the figures for the Mott-Schottky curves, the potential was scanned from −0.2 to 0.8 V (versus Ag/AgCl) at various scan rates in different concentrations of solutions (Methanol, Ethanol, Urea). Electrochemical impedance spectroscopy (EIS) graphs with a frequency range of 100 kHz to 10 Hz and modulation amplitude of 5 mV were taken for these concentration measurements at an open circuit voltage (0.5 V vs. Ag/AgCl). Polarization curves obtained using linear sweep voltammetry (LSV) at RT with a 10 mV/s scan rate were used to assess the electrocatalytic activity of the samples.

## 3. Results and Discussion

### 3.1. Morphological Study

**Figure 2:**
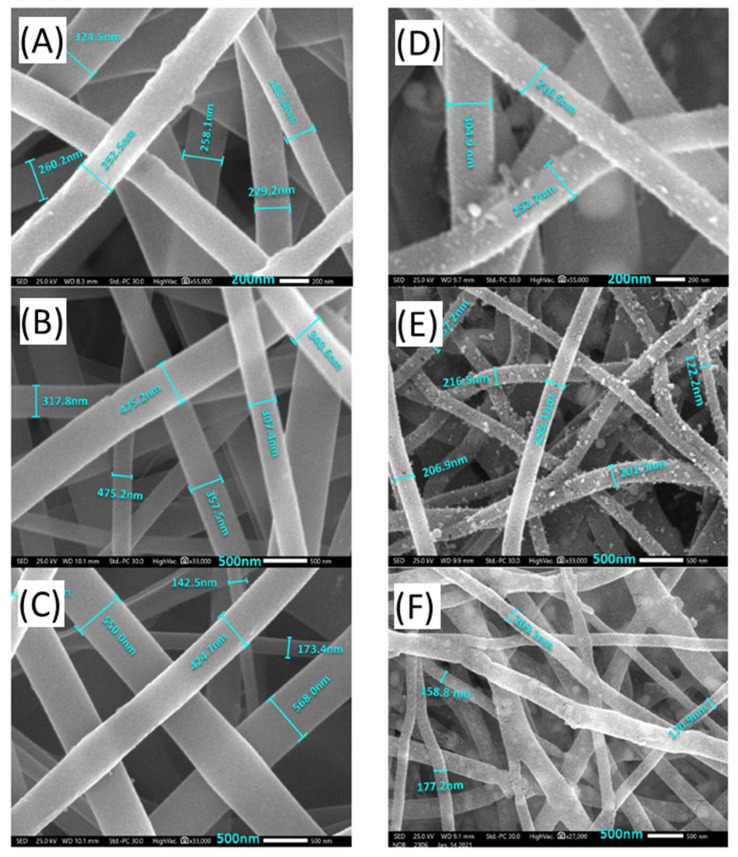
SEM images for (**A**–**C**) PVA/NiAc/CoAc/H_2_PtCl_6_ nanofibers mats before carbonization and (**D**–**F**) carbon nanofibers loaded with Ni_4_Co_2_Pt, Ni_5_CoPt and Ni_6_Pt nanoparticles (after carbonization).

The electrospinning process was used to successfully produce PVA/NiAc-CoAc-Ptcl nanofibers. Figure 2A–C shows SEM images for the PVA nanofibers mats loaded with the salts of the metals (NiAc-CoAc-PtCl) before carbonization. All of the SEM images of the nanofibers obtained indicate clearly smooth, bead-free nanofibers. The average fiber diameter ranges from 200 to 580 nm. No significant difference was observed between the fiber morphologies of the prepared nanofibers. After the carbonization process, the polymer is transformed into carbon nanofibers [50], with metallic nanoparticles being distributed over them. Figure 2D–F shows SEM images of carbon nanofibers loaded with Ni_4_Co_2_Pt, Ni_5_CoPt, and Ni_6_Pt nanoparticles after carbonization at 900 °C in an argon atmosphere. These images demonstrate that the used approach for making decorated carbon nanofibers with metallic nanoparticles can be employed successfully. Except for reducing the fiber diameter to the range of 150–200 nm, the carbonization procedure had no significant influence on the nanofibrous shape of the electrospun nanofibers, as shown in the figure.

**Figure 3:**
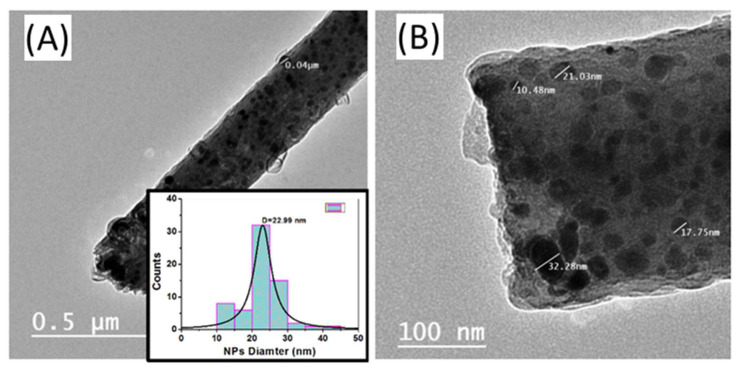
(**A**,**B**) TEM images with different magnifications for the sample Ni_5_CoPt/CNFs. The inset in (**A**) shows the corresponding particle diameter distribution of Ni_5_CoPt NPs on the surface of the CNFs.

The size and distribution of the metallic nanoparticles on the carbon nanofibers can be examined using a transmission electron microscope (TEM). Figure 3 shows TEM images with different magnifications for the sample S2 (Ni_5_CoPt/CNFs), as an example. These TEM images demonstrated that the Ni_5_CoPt NPs were almost homogeneously dispersed in the CNFs and evenly grown on the surface of CNFs. The inset of Figure 3A shows the nanoparticle diameter distribution. The average diameter of the NPs was ~23 nm.

### 3.2. Elemental Analysis

**Figure 4:**
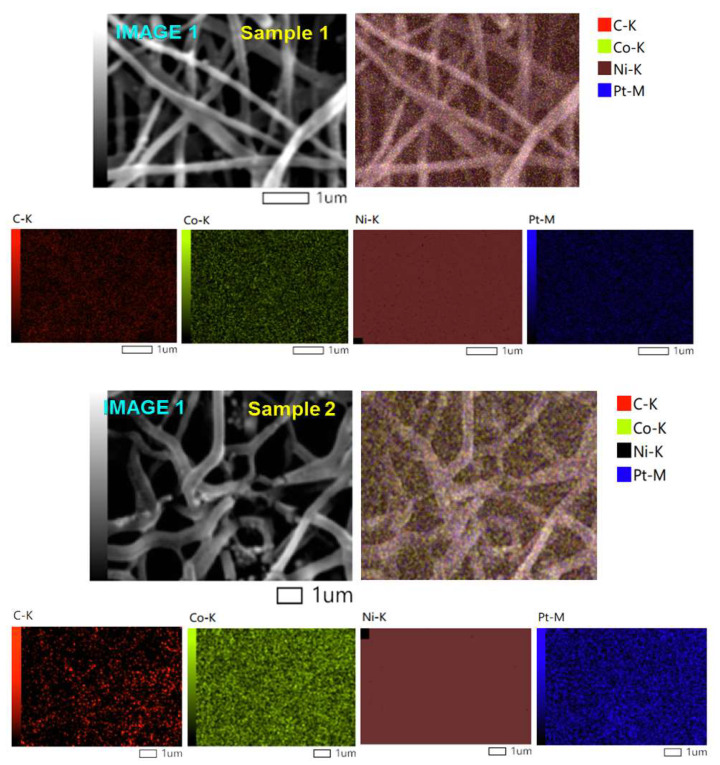
The SEM images and corresponding EDX mapping analysis of sample Ni_4_Co_2_Pt/CNFs (S_1_) and sample Ni_5_CoPt/CNFs (S_2_).

Figure 4 demonstrates the SEM image and corresponding EDX mapping analysis of the Ni_4_Co_2_Pt/CNFs (sample 1) and Ni_5_CoPt/CNFs (sample 2), respectively. (IMG1) represents the area that was scanned to know the components of the fibers. From Figure 4, for sample 1 and sample 2, it can be seen that the distribution of C element as nanofibers was homogeneous and dense on the whole samples. Also, the nanoparticles of Ni, Pt and Co are distributed fully and uniformly on the surface of carbon nanofibers, as presented in the figure. Furthermore, the elemental mapping images (Figure 4) confirmed the shiny NPs as Ni, Pt and Co no other foreign impurities were detected, and this was also proven by the X-ray results. According to this study, transition metals (nickel, cobalt and platinum) were successfully integrated into carbonized PVANFs. This is owing to the transition metal’s ability to bind to PVA’s hydroxyl groups and then the carbon content after carbonization [51]. The proposed preparation process is considered a good procedure for the synthesis of the carbon nanofibers decorated by metal nanoparticles after carbonization.

**Figure 5:**
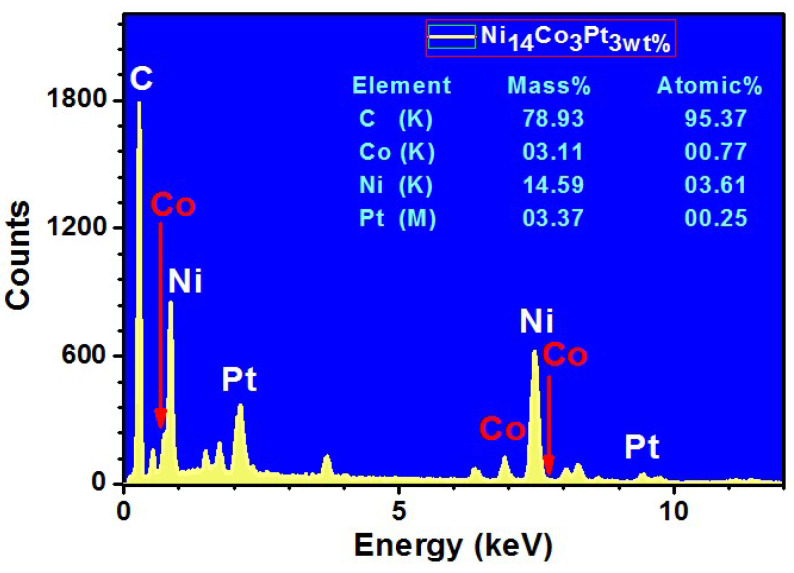
EDX spectrum for sample S2, Ni_5_CoPt/CNFs.

The chemical content of the sample Ni_5_CoPt/CNFs (sample S2) was identified through EDX analysis (Figure 5). The mass and the atomic percentages of its elements are listed in the inset of Figure 5. The mass percentages of C, Ni, Co and Pt are 78.93%, 14.59%, 3.11% and 3.37%, while the expected values are 80%, 14%, 3% and 3%, respectively. The small difference between the expected and produced ratios could be attributed to experimental circumstances.

### 3.3. Structure Analysis (XRD)

**Figure 6:**
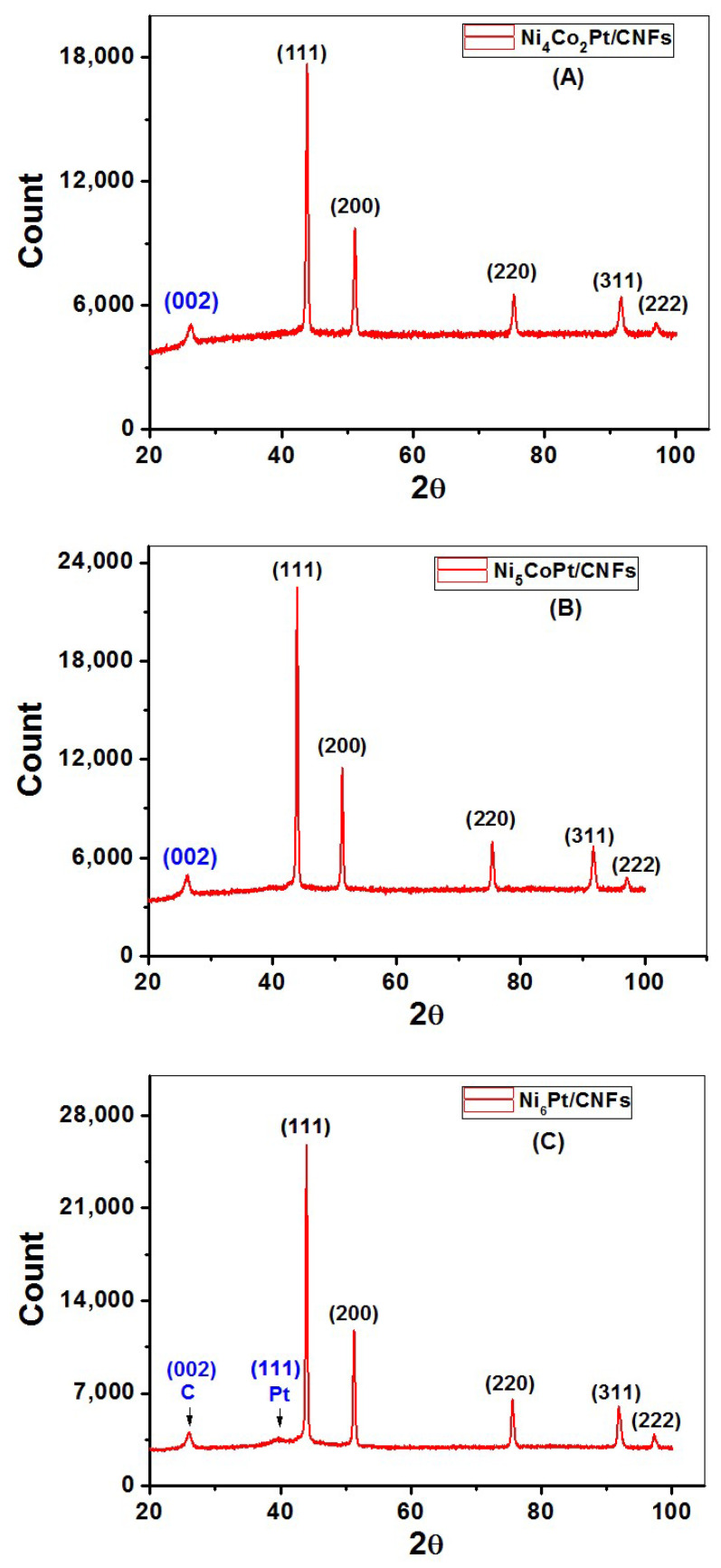
X-ray diffraction patterns of (**A**) Ni_4_Co_2_Pt/CNFs, (**B**) Ni_5_CoPt/CNFs, and (**C**) Ni_6_Pt/CNFs.

The XRD technique has been extensively used to determine the phases and crystallinity in materials. XRD patterns were recorded, as shown in Figure 6A–C, five diffraction peaks for Ni_4_Co_2_Pt/CNFs, Ni_5_CoPt/CNFs, and Ni_6_Pt/CNFs, are characteristic of a face-centered cubic (FCC) lattice. The broad peak at approximately 26° relates to the (002) plane of carbon (JCPDS; 41-1487) [52,53]. The CNFs were graphitic and this would increase the conductivity of the prepared electrodes [54]. The amorphous nature of PVA and the occurrence of sharp peaks connected to metallic NPs [55] could explain the low intensity of this peak. The slight change in the peak location of the prepared sample to higher angles compared to the data in (JCPDS file no. 04-0850) [56,57] for Ni indicates the formation of the ternary alloys [58]. XRD analysis confirmed the carbonization of the prepared nanofibers. Only one Pt peak appears in sample S_3_ as shown in Figure 6C. For cobalt, its diffraction angles match that of nickel, so the peaks may be convoluted with nickel peaks due to its high proportion compared to cobalt. In contrast, the absence of amorphous or crystalline Pt and Co phases suggests that all metals are integrated into the FCC Ni phase; resulting in a real alloy at all particle sizes. The XRD analysis confirms that the nanoparticles exist only in the metallic phase in the composite no hydroxide or oxide phases are noticed in XRD patterns.

The particle size was calculated and listed in Table 2 using the Debye-Scherer equation with data from the XRD analysis. The most intense diffraction peak (111) was taken for the determination of the particle size. The strain-free Scherer equation is in the form:

(1) $$D=\frac{K\lambda }{\beta cos\theta }$$

where *K* = 0.9, *λ* = 0.154 nm, *β*, is the full width at high maximum (FWHM) and *θ* is the peak position. From Table 2 it can be noticed that the calculated particle size of the prepared alloys is relatively large. The increase in the particle size of the prepared samples may be due to that, the carbonization process was carried out at a high temperature may lead to the MNPs agglomerating with each other. From the table, it can also be noted that the values of the crystal lattice constant increase slightly with the increase in the proportion of cobalt due to the difference in the size of the cobalt atom from nickel.

The surface area (SA) is defined as the total surface area of a solid material per unit of mass. The surface area (SA) values can be determined according to the following equation [59,60]:

(2) $$SA=\frac{6000}{D\rho }$$

where *D* is the particle size in nm obtained from X-ray data and *ρ* is the density of the alloy. The XRD-determined surface area values for the samples S_1_, S_2_ and S_3_ are listed in Table 2. SA values for the prepared alloys are smaller than those for the commercial Pt catalyst but larger than that of Pd-Co-Mo alloy on activated carbon heated at 900 °C [59] as shown in Table 2. For Ni systems, the carbonization temperature of 900 °C may be too high, inducing atom agglomeration and resulting in excessive grain size.

### 3.4. Electro-Oxidation Measurements

#### 3.4.1. Effect of Sample Composition and Electrolyte

**Figure 7:**
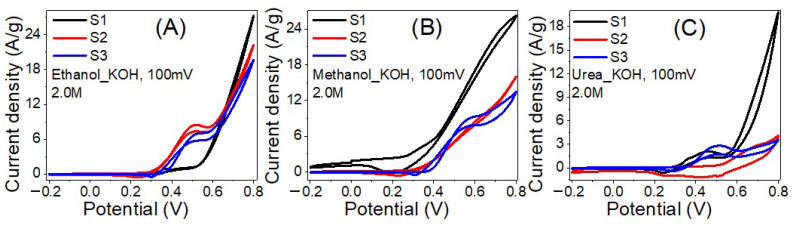
The influence of the sample composition on the EC catalytic activities for oxidating 2.0 M (**A**) ethanol, (**B**) Methanol and (**C**) Urea in 1 M KOH solutions at RT (20 °C).

The EC measurements were quantified utilizing CHI 660E Electrochemical workstation attached with a 3-electrode EC cell of Ag/AgCl reference electrode, glassy carbon electrode coated by the fabricated nanofibers (working electrode), and a platinum wire (counter electrode). Figure 7 shows the effect of the sample composition on the EC catalytic activities for oxidating 2.0 M (A) ethanol, (B) methanol and (C) urea in 1 M KOH solution at RT (20 °C) and scan rate of100 mV/s. In 2.0 M ethanol, as the Ni% decreased from 17% to 11% and the Co% increased from 0 to 6%, Figure 7A. The incorporation of Co significantly improves the EC catalytic activity in terms of current density, increasing the maximum current density from 19.7 to 27.3 A/g. The current density of the oxidizing peak is 0.9 A/g @ 0.37 V, 8.2 A/g @ 0.52 V and 7.4 A/g @ 0.53 V for samples with Ni% of 11% (S1), 14% (S2) and 17% (S3). Also, the onset potentials are 0.30, 0.33, and 0.35 V, respectively.

In 2.0 M methanol, Figure 7B, the incorporation of Co considerably improves the EC catalytic activity by increasing the maximum current density from 13.4 to 26.2 A/g as the Co ratio increased from 0 to 6%. The current density of the oxidation peak is increased from1.5 A/g @ 0.32 V to 9.4 A/g @ 0.58 V as the Ni% increased from 11% to 17% with onset potentials of 0.26 and 0.37 V, respectively. This means 6.3 folds enhancement in the oxidation current by increasing Ni%. Furthermore, whereas the methanol EC oxidation peak is evident in samples S1 and S3, it is not apparent in sample S2.

In 2.0 M urea, Figure 7C, the maximum current density was raised from 3.6 to 19.8 A/g as the Co ratio increased from 0 to 6%. The current densities of the oxidation peaks are 1.2 A/g @ 0.36 V, 2.6 A/g @ 0.69 V and 2.8 A/g @ 0.52 V for Ni% of 11%, 14% and 17% with onset potentials of 0.31, 0.51 and 0.36 V, respectively. The observed results are intriguing because they show a possible rise in the EC catalytic activity of the suggested catalyst in terms of the amount of ethanol, methanol, and urea oxidized on its surface. This finding is more pronounced with methanol and ethanol than urea electrolysis. Based on the data, the insertion of Co with Ni could positively reduce the required activation energies of the oxidation reactions. Moreover, the effect of the electrolyte can be observed from Figure 7, whereas the current density for the sample with 6% Co doping has the order: ethanol > methanol > urea. The observed onset potentials for methanol are lower than ethanol and urea.

#### 3.4.2. Effect of Electrolyte Concentration

**Figure 8:**
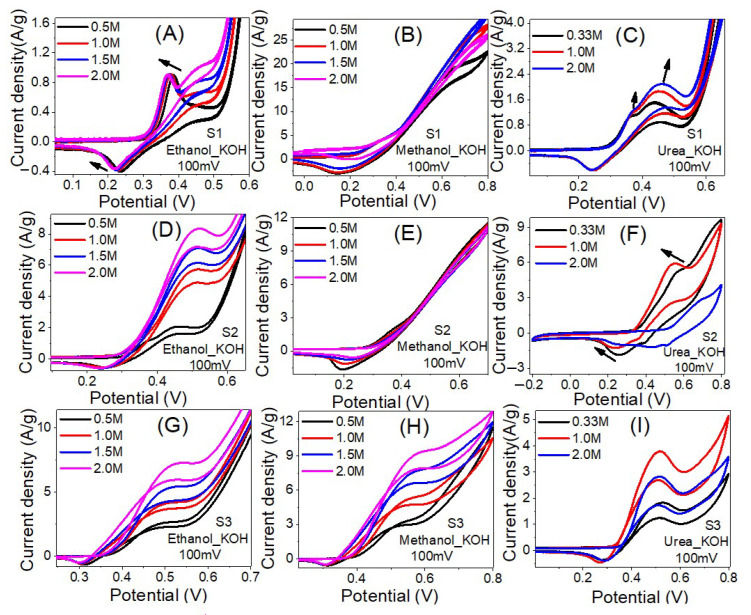
The influence of ethanol, Methanol, and Urea concentration on the electrocatalytic activity of samples S1 (**A**–**C**), S2 (**D**–**F**) and S3 (**G**–**I**) in 1.0 M KOH solution at RT.

Ni is believed to be the best EC catalyst for the oxidation of ethanol and methanol [61]. Figure 8 shows CV voltammograms of the prepared nanofibers samples at different ethanol, methanol, and urea concentrations. As shown in Figure 8A, as the ethanol concentrations increase the current densities of the oxidation peaks are increased and accompanied by a shift to a lower potential position. The maximum current density was increased from 26.6 to 28.2 A/g as the ethanol concentration increased from 0.5 M to 1.5 M, and then slightly decreased to 27.3 A/g as the concentration increased to 2.0 M. The current densities of the oxidation peaks are changed from 0.85 A/g @ 387 mV to 0.90 A/g @ 368 mV with onset potentials reduced from 315 to 298 mV as the ethanol concentration changed from 0.5 M to 2.0 M. This proves the enhancement of the EC catalytic activities of sample S1 to ethanol electrooxidation as its concentration increased. The ethanol electrooxidation process is based on the adsorption of the intermediate components and reactants, which is followed by dissociation stages. The O–H bond was first broken in the ethanol dehydrogenation, resulting in the formation of ethoxy species (CH_3_CH_2_O). These species were then transformed to acetaldehyde (CH_3_CHO). Many processes oxidize the produced acetaldehyde, resulting in acetate ions (CH_3_COO^−^), acetyl (CH_3_CO), methane, acetone (CH_3_COCH_3_), crotonaldehyde (CH_3_CHCHCHO), other hydrocarbons, carbonate ion (CO_3_^2^^−^), CO_2,_ and CO [62,63].

Similar results are observed for the electrooxidation of methanol and ethanol using sample S1 as shown in Figure 8B,C. For methanol, the values of the maximum current density were 22.7, 28.3, 30.6 and 26.2 A/g at methanol concentrations of 0.5 M, 1.0 M, 1.5 M and 2.0 M, individually. The current densities of the oxidation peaks are changed from 2.2 A/g @318 mV to 2.8 A/g @318 mV with onset potentials increased from 219 to 235 mV as the methanol concentration changed from 0.5 M to 1.5 M.

For urea, the recorded maximum current densities were 21.2, 20.7, 19.8 A/gat urea concentrations of 0.33 M, 1.0 M and 2.0 M i.e., as the concentration increased, the maximum current density decreased. Additionally, two oxidation peaks were observed as indicated by the arrows in Figure 8C. The current density of the first oxidation peak is changed from 1.0 A/g @ 358 mV to 1.2 A/g @ 364 mV with onset potentials increased from 303 to 308 mV as the urea concentration changed from 0.33 M to 2.0 M. The current density of the second oxidation peak is changed from 1.4 A/g @ 430 mV to 2.0 A/g @ 451 mV with onset potentials reduced from 279 to 294 mV as the urea concentration changed from 0.33 M to 2.0 M.

For Sample S2, Figure 8D–F, the highest current density was observed using 2 M ethanol. The value of the highest current density is 21.9 A/g @ 2 M ethanol, 15.3 A/g @ 2 M methanol and 9.7 A/g @ 0.33 M urea. For ethanol, the current density of the oxidation peak is increased from 1.1 A/g @ 449 mV to 8.2 A/g @ 515 mV with onset potential increased from 310 to 329 mV as the concentration increased from 0.5 M to 2.0 M. For methanol, the current density of the oxidation peak is decreased from 1.5 A/g @ 356 mV to 1.1 A/g @ 359 mV as the concentration increased from 0.5 M to 2.0 M with onsets increased from 280 to 298 mV. For urea, the oxidation peak current density is increased from 1.8 A/g @ 560 mV to 6.0 A/g @ 545 mV with increasing the concentration from 0.33 M to 1.0 M and then decreased to 2.5 A/g @ 688 mV as the concentration raised to 2.0 M. Similarly, the inset potential reduced from 357 to 353 mV and then increased to 522 mV. Therefore, this sample is more efficient for electrooxidation of 2.0 M ethanol and 1.0 M urea than 2.0 M methanol. Whereas the energy required for the electrooxidation of urea is less than ethanol.

For Sample S3, Figure 8G–I, the highest current density was observed using 2 M ethanol. The value of the highest current density is 19.1 A/g @ 2 M ethanol, 12.9 A/g @ 2 M methanol and 5.2 A/g @ 1.0 M urea. For ethanol, the current density of the oxidation peak is increased from 2.7 A/g @ 508 mV to 7.4 A/g @ 530 mV with onset potential decreased from 377 to 354 mV as the concentration increased from 0.5 M to 2.0 M. For methanol, the current density of the oxidation peak is increased from 3.0 A/g @ 508 mV to 9.4 A/g @ 584 mV as the concentration increased from 0.5 M to 2.0 M with onsets decreased from 403 to 372 mV.

For urea, the oxidation peak current density is increased from 1.7 A/g @ 528 mV to 3.7 A/g @ 514 mV with increasing the concentration from 0.33 M to 1.0 M and then decreased to 2.7 A/g @ 511 mV as the concentration raised to 2.0 M. The inset potential declined from 369mV @ 0.33 Mto 339 mV @ 1.0 M and then increased to 366 mV @ 2.0 M. Therefore, this sample is more efficient for electrooxidation of 2.0 M methanol and 2.0 M ethanol than 1.0 M urea. The energy required for the electrooxidation follows the order urea < ethanol < methanol. For the urea electrooxidation behaviors on the S1, S2 and S3 catalysts, Figure 8C,F,I, there are a pair of distinct redox current peaks, which correspond to the reversible variation between Ni^2+^ and Ni^3+^, based on Equation (3) [64].

Ni(OH)_2_ + OH^−^ ←→ NiOOH + H_2_O + e^−^ (3)

In the forward scan, the observed anodic peak is ascribed to the oxidation of Ni(OH)_2_ to NiOOH [65]. Whereas, during the reverse scan, the cathodic peak refers to the reduction of NiOOH to Ni(OH)_2_ [66]. The second anodic peak observed for S1 is appeared due to the transformation of Co(OH)_2_ to CoOOH. In summary, the optimized concentrations for all samples and electrolytes are reported in Table 3.

#### 3.4.3. Effect of the Scan Rate

**Figure 9:**
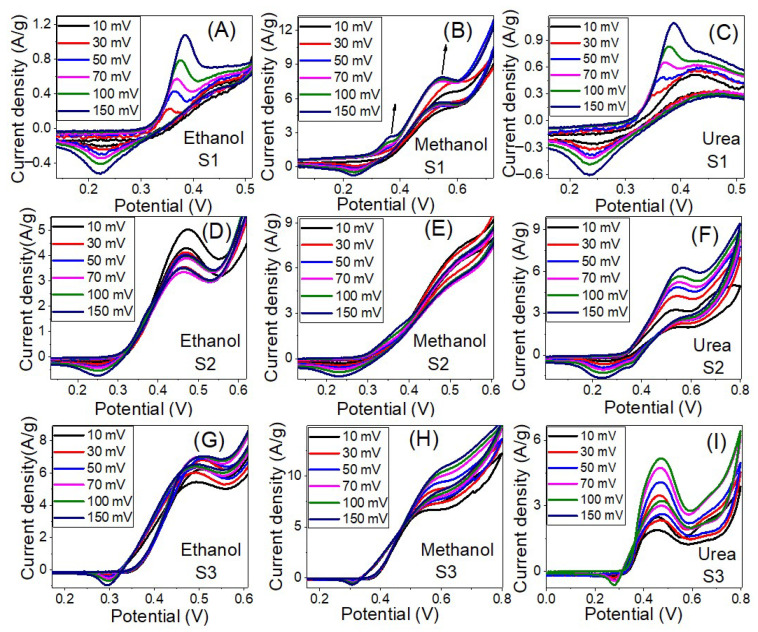
The influence of the scan rate on the electrocatalytic activity of samples S1 (**A**–**C**), S2 (**D**–**F**) and S3 (**G**–**I**) for electrooxidation of ethanol, Methanol, and Urea in 1.0 M KOH solution at RT.

**Figure 10:**
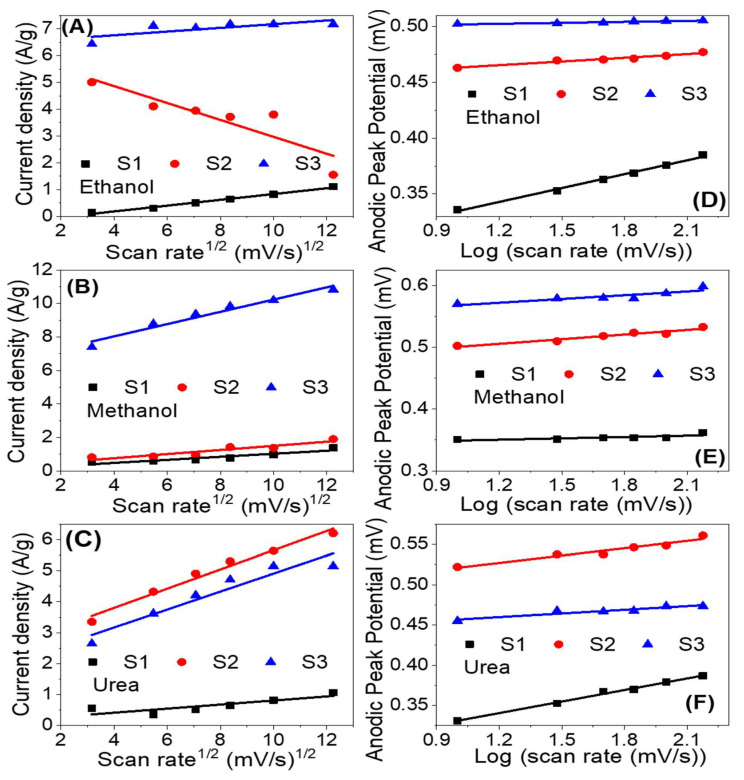
Current density versus the square root of the scan rate(**A**–**C**) and anodic peak potential versus the logarithm of the scan rate (**D**–**F**) for the electrooxidation of ethanol, methanol, and urea utilizing samples S1, S2 and S3.

Figure 9 shows the influence of the scan rate on the electrocatalytic activity of the prepared samples for electrooxidation of ethanol, methanol and urea in 1.0 M KOH solution at RT. As the scan rate increased the current density of the oxidation peak increased and the peak position shifted to a higher potential. The roughly linear plots between ethanol, methanol and urea oxidation peaks current density and the square root of the scan rate, as shown in Figure 10A–C, suggest a diffusion-controlled mechanism [67,68]. The diffusion-controlled characteristics are further clarified using the plot of log (anodic current density) vs. log (scan rate), as shown in Figure S2 (supplementary data) i.e., these data demonstrate that the oxidation process is diffusion-controlled [69]. The increase of the oxidation current density with increasing the scan rate points to improve the kinetic oxidation of methanol. Whereas the linear dependence of the anodic peak potential vs. the logarithm of the scan rate, Figure 10D–E, indicates kinetics limitations for the reaction [70,71]. The peak height is proportional to the square root of the potential scan rate when the positions of the anodic peaks do not change with the potential scan rate, thermodynamically reversible electrochemical reactions, according to the following equation (T = 25 °C): IP = (2.69 × 10^5^) n^3/2^AD^1/2^ *v*^1/2^C. Whereas IP is the peak current, n is the number of electrons per reactant molecule, A is the electrode area, *v* is the potential scan rate, D is the diffusion coefficient, and C is the bulk concentration of the reactant [72]. Note that the electrochemical reactions are not completely reversible according to Figure 9 because the anodic peak potential becomes more positive, and the cathodic peak potential becomes more negative as the scan rate increases. Therefore, the separation of the anodic and cathodic peaks becomes larger than in the reversible case.

The anodic current increases progressively with increasing scan rates (range from 10 to 150 mV s^−1^), as seen in Figure 9C,F,I). In addition, at higher scan rates, the shift of anodic peak potentials to a more positive orientation (Figure 9F) displays a change in the reaction kinetics between urea and Ni^3+^ [67]. The shift in peak potentials toward higher positive values can be attributed to the selective adsorption of urea on Ni^3+^ sites. Furthermore, the linear relationship between the anodic peak current density and the square root of scan rate (*v*^1/2^) implies that the electrooxidation reaction on the catalytic samples S1–S3 is controlled by diffusion (Figure 10A–C) [68,70,73]. The anodic peak potential increases approximately linearly with the logarithm of the scan rate (log *v*) in Figure 10D–F, showing that ethanol, methanol, and urea electrooxidation have kinetic constraints [70]. According to these, the electrooxidation of ethanol, methanol, and urea on our catalysts is controlled by a combination of diffusion and kinetic limitations [74].

#### 3.4.4. Linear Sweep Voltammetry

**Figure 11:**
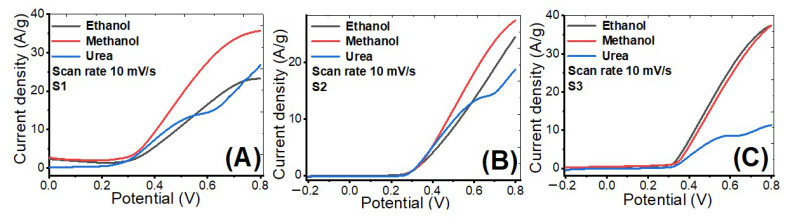
Linear sweep voltammetry plots at scan rate 10 mV/s of prepared (**A**) S1, (**B**) S2 and (**C**) S3 in ethanol, methanol, and urea at the optimized concentrations.

Figure 11A–C shows the LSV curves for the prepared samples (S1, S2, S3). The LSV curves were measured by a three-electrode cell in 1 M KOH at optimal ethanol, methanol, and urea concentrations. The scanning rate of LSV is performed at 10 mV/s in the potential range from −0.2 to 0.8 V. As shown in the supplementary data (Table S1), this voltage window has been employed in many earlier works for ethanol, methanol, and urea electro-oxidation. For sample S1, the current density increased from 23.2 to 26.8 and then to 35.7 A/g @ 799 mV for methanol, urea, and ethanol, respectively. For sample S2, the current density increased from 18.7 to 24.5 and then to 27.4 A/g @ 799 mV for urea, methanol, and ethanol, respectively. For sample S3, the current density increased from 11.3 to 37.40 and then to 37.42 A/g @ 799 mV for urea, methanol, and ethanol, respectively. When compared to urea and methanol solutions, the ethanol solution has the highest current density at a potential of 799 mV for all samples. Also, sample S2 has the lowest current density compared with other samples, which can be attributed to the comparatively large particle size and small surface area, and lower content of nickel compared to S1.

To investigate the kinetics of electrochemical reactions, the Tafel curves were used (Figure S3, supplementary data). The corresponding Tafel slopes were presented in Table 3. For methanol, the slopes are lower. Also, the Tafel slope is reduced from 29.6 ± 0.1 to 19.1 ± 0.2 mV/dec for ethanol, from 30.6 ± 0.1 to 22.6 ± 0.2 mV/dec for methanol, and from 38.5 ± 0.2 to 23.6 ± 0.2 mV/dec for urea by increasing the Ni% from 11 to 17%, as shown in Table 3. It is clear that S1 has a slightly higher Tafel slope than S3.

#### 3.4.5. Stability Study

**Figure 12:**
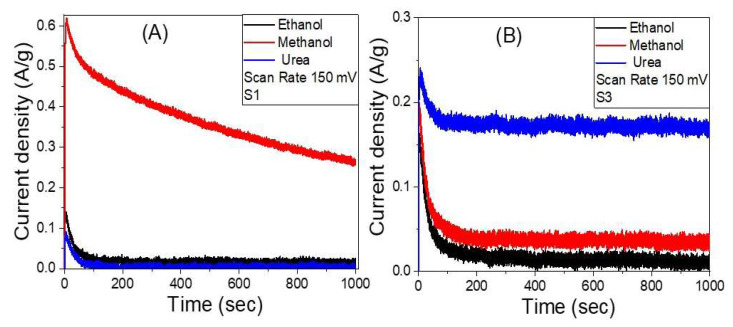
The variation of the current density versus time, chronoamperometry measurements, for samples (**A**) S1 and (**B**) S3 in ethanol, methanol, and urea.

The stability of the samples S1 and S3 in ethanol, methanol, and urea at 0.8 V between the working electrode and the Pt counter electrode is examined for an extended period. The fluctuations of current density versus time, as measured by chronoamperometry, are shown in Figure 12A,B. Sample S1 showed higher stability toward the methanol electrooxidation than ethanol and urea electrooxidation. The current density is decreased from 0.61 A/g to 0.26 A/g for methanol as the time reaction increased from 5 s to 1000 s before reaching a stable production rate, which is attributed to a limited corrosion process that occurs between the electrode and the redox electrolyte [75]. Sample S3 showed higher stability toward the urea electrooxidation than ethanol and ethanol electrooxidation. The current density is decreased from 0.24 A/g to 0.17 A/g for urea as the time increased from 7 s to 1000 s before reaching a stable production rate. This demonstrates that the S3 electrode has great chemical stability and a long lifetime as a working electrooxidation electrode, despite the initial drop in current density i.e., these findings suggest that using Co as a co-catalyst to boost the electrocatalytic activity of Ni/Ag catalysts has significant benefits.

#### 3.4.6. Electrochemical Impedance Spectroscopy (EIS)

**Figure 13:**
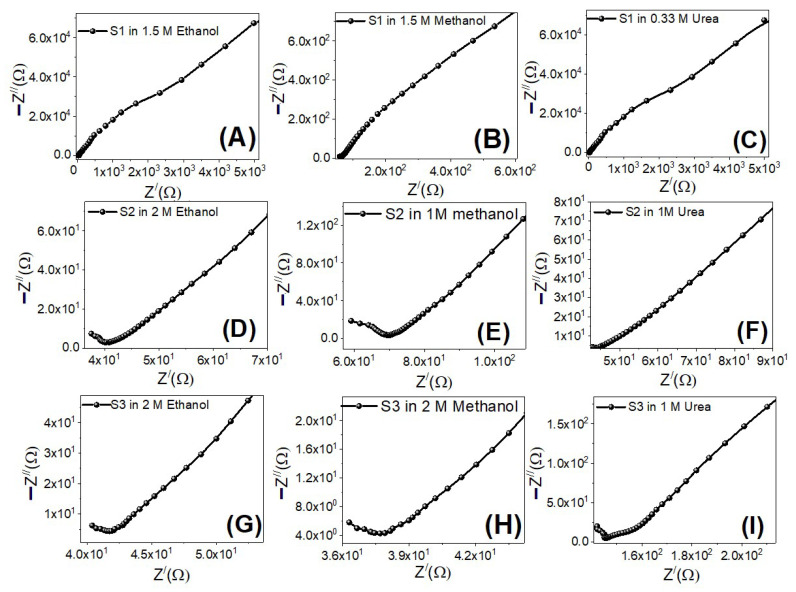
Nyquist plots for electrodes S1 (**A**–**C**), S2 (**D**–**F**) and S3 (**G**–**I**) in ethanol, methanol, and urea at the optimized concentrations and 0 V (vs. Ag/AgCl).

The electrooxidation catalytic efficiency of the working electrodes is largely determined by charge carrier dynamics. EIS experiments were carried out at RT utilizing an electrochemical workstation to explore the charge carrier dynamics of the samples (CH Instruments CHI660E). EIS is a valuable approach for evaluating the electrocatalyst’s interfacial characteristics [76]. The sum of real, Z′, and imaginary, Z″, components contributed by the cell’s resistance and capacitance is the impedance [77]. EIS measurements were done at 0 V (vs Ag/AgCl) under illumination with a frequency range of 0.01–100,000 Hz with the S1–S3 electrodes immersed in ethanol, methanol, and urea electrolytes. The Nyquist plots for all electro-electrodes are displayed in Figure 13. As shown in Figure 13A–C for sample S1, all working electrodes showed a semicircle at high frequencies due to charge transfer processes at electrode/electrolyte boundaries (charge transfer resistance), and two straight line segments with slopes of 45° and 55° at low frequencies due to diffusion-controlled processes (Warburg impedance) and additional limited capacitive behavior (double-layer capacitance) i.e., Mixed diffusion, and kinetic controlled routes are depicted in the EIS data. In comparison to the other electrodes, electrode S1 has the lowest charge transfer resistance and electrolyte resistance, improving the electrooxidation catalytic process.

**Figure 14:**
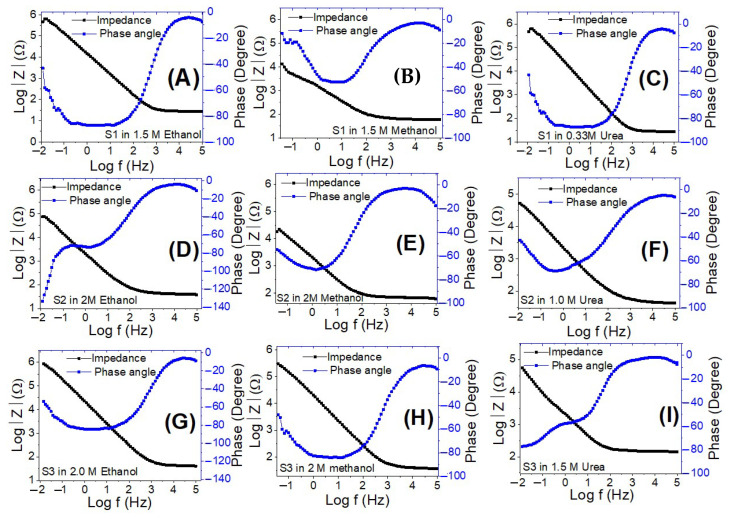
Bode plots for electrodes S1 (**A**–**C**), S2 (**D**–**F**), and S3 (**G**–**I**) in ethanol, methanol, and urea at the optimized concentrations and 0 V (vs. Ag/AgCl).

Figure 14 shows Bode charts for all electrodes using ethanol, methanol, and urea at 0 V (vs. Ag/AgCl) at RT. Figure 14 depicts the variation of the logarithm of the total impedance (Z) with the frequency logarithm, as well as the variation of the phase with the frequency logarithm. Plotting the Log (Z) with Log (*f*) reveals a resistive regime at low frequencies due to the charge transfer resistance, as well as a very minimal capacitive contribution at high frequencies due to the electrode’s double-layer capacitance [78]. With rising Co levels, there is a significant reduction in charge recombination at the electrolyte/electrode interfaces. This also includes a kinetically simple electroxidation process, increased ionic conductivity, and electrolytes diffusion across sample S1. As a result, when compared to the other electrodes, this electrode demonstrated superior electrooxidation catalytic performance.

## 4. Conclusions

In this work, NiCoPt/CNFs nanoparticles were successfully loaded on carbon nanofibers utilizing electrospinning followed by carbonization at 900 °C for 7 h in an argon atmosphere. The chemical composition: structures, nanomorphologies, and electrochemical properties were investigated utilizing different analysis techniques. The carbonization process of the obtained electrospun nanofibers reduces the fiber diameter from 200–580 nm to 150–200 nm for samples with varying Co percent (0–6%). The EDX mapping revealed a homogeneous and uniform distribution of Ni, Pt and Co on the carbonized PVANFs’ surface. The Ni-Co-Pt/CNFs have a face-centered cubic (FCC) lattice with crystallite sizes decreasing from 24.93 to 22.57 nm as the cobalt ratio increased. The electrocatalytic properties of the samples were investigated for electrooxidation of ethanol, methanol, and urea. The effects of electrolyte concentration, scan rate, electrode stability, and EIS spectroscopy were studied. Inserting Co as a co-catalyst with nickel could successfully lower the electrooxidation reaction’s required activation energy and increase the electrode’s stability. Moreover, in 2 M electrolyte concentration, the current density for the 6% Co sample is in the following order: J*_ethanol_* > J*_methanol_* > J*_urea_*. The Ni_5_CoPt/CNFs electrode in 1.5 M methanol had the lowest onset potential, while the maximum current densities were 28.2, 30.6 and 21.2 A/g for 1.5 M ethanol, 1.5 M methanol and 0.33 M urea, respectively. This study demonstrated a novel approach to creating a highly active Ni-Co-Pt-based electrocatalyst oxidation of ethanol, methanol, and urea.

## Figures and Tables

**Table 1:** Metal composition of the electro-catalysts in total metal loading of 20 wt%.

Catalyst Name	Catalyst Molar Ratio	Ni, wt%	Co, wt%	Pt, wt%	Metal wt%
S1	Ni_4_Co_2_Pt/CNFs	11	6	3	20
S2	Ni_5_CoPt/CNFs	14	3	3	20
S3	Ni_6_Pt/CNFs	17	0	3	20

**Table 2:** Particle sizes were calculated from the XRD results using the Scherer equation.

Sample	(hkl)	Peak Position	FWHM (β) (O)	D (nm) Scherer	SA (m^2^/gm)	(a) Lattice Parameter (nm)
S1	(111)	43.876	0.3794	22.57	23.0	0.3570
S2	(111)	43.920	0.3483	24.59	21.1	0.3567
S3	(111)	43.979	0.3436	24.93	20.7	0.3562
Pd-Co-Mo/AC ref. [59] At 900 °C (70:20:10) nanoparticles				41.2	13.0	
Commercial Pt/AC As received				3.32	85.7	

**Table 3:** Optimized concentrations and Tafel slope values for S1, S2 and S3 in ethanol, methanol, and urea electro-oxidation reactions.

Parameter	Optimized Concentration			Tafel Slop (mV/Dec)		
Sample	S1	S2	S3	S1	S2	S3
Ethanol	1.5	2	2	29.6 ± 0.1	25.6 ± 0.1	19.1 ± 0.2
Methanol	1.5	2	2	30.6 ± 0.1	28.5 ± 0.1	22.6 ± 0.2
Urea	0.33	1	1	38.5 ± 0.2	31.9 ± 0.3	23.6 ± 0.2

## Data Availability

Not applicable.

## Supplementary Materials

The following supporting information can be downloaded at: https://www.mdpi.com/article/10.3390/nano12030492/s1. Figure S1: Plots of Log (Current density) versus Log (scan rate) for the electrooxidation of (A) ethanol, (B) methanol and (C) urea utilizing samples S1, S2 and S3; Figure S2: Plots of overpotential versus log (current density) for calculating the Tafel slopes of the prepared (A) S1, (B) S2 and (C) S3 in ethanol, methanol, and urea at the optimized concentrations; Table S1: Voltage windows, electrocatalysts, and used solutions in previously reported works relative to the present study for ethanol, methanol, and urea electro-oxidation. References [79,80,81,82,83] are cited in the supplementary materials.
