# Supplementary material for: Synthesis and Characterization of NiCoPt/CNFs Nanoparticles as an Effective Electrocatalyst for Energy Applications
Source: Nanomaterials (Basel). 2022 Jan 30;12(3):492. doi: 10.3390/nano12030492 (PMC8840489; doi:10.3390/nano12030492)
Supplement: Supplementary file 1 [file nanomaterials-12-00492-s001.zip › nanomaterials-1505563-supplementary.pdf]

nanomaterials

Supplementary data

Synthesis and Characterization of NiCoPt/CNFs Nanoparticles as an Effective Electrocatalyst for Energy Applications

E.E. Abdel-Hady [1,2], Mohamed Shaban [3,4,*], M.O. Abdel-Hamed [1,2], Ahmed Gamal [4], Heba Yehia [1] and Ashour M. Ahmed [4]

1 Physics Department, Faculty of Science, Minia University, Minia 61519, Egypt; esamhady@yahoo.com (E.E.A.-H.); mazosman2005@yahoo.com (M.O.A.-H.); hdody393@gmail.com (H.Y.)
2 Academy of Scientific Research and Technology (ASRT) of the Arab Republic of Egypt, Cairo 11516, Egypt
3 Department of Physics, Faculty of Science, Islamic University in Madinah, Al-Madinah Al-Munawarah 42351, Saudi Arabia; mssfadel@aucegypt.edu (M.S.)
4 Nanophotonics and Applications (NPA) Lab, Physics Department, Faculty of Science, Beni-Suef University, Beni-Suef 62514, Egypt; mssfadel@aucegypt.edu (M.S.); a_gamal21@yahoo.com (A.G.); ashour.elshemey@gmail.com (A.M.A.).
* Correspondence: mssfadel@aucegypt.edu

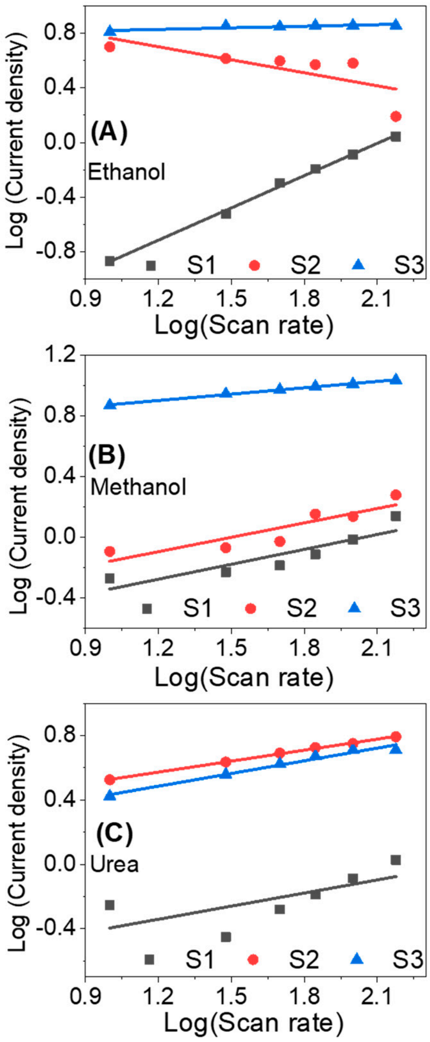

Figure S1. Plots of overpotential versus log (current density) for calculating the Tafel slopes of the prepared (A) S1, (B) S2, and (C) S3 in ethanol, methanol, and urea at the optimized concentrations.

Table S1. Voltage windows, electrocatalysts, and used solutions in previously reported works relative to the present study for ethanol, methanol, and urea electro-oxidation.

Ref	Electrocatalyst	Solution	Voltage window
1	Ni-Sn/C	Methanol	0 to 1 V versus Ag/AgCl
2	$NiCo_2O_4$	Methanol	−0.2 to 0.6 V versus Ag/AgCl
3	Pt-Sn	Ethanol	−0.2 to 1 V versus Ag/AgCl
4	NiSn / C	Urea	0 to 1 V versus Ag/AgCl
5	W- Ni/C	Ethanol	0 to 1 V versus Ag/AgCl
Present work	$Ni_{17-x}Co_xPt_3$ (x= 0 to 6 wt%)	Ethanol, Methanol, and Urea	−0.2 to 0.8 V versus Ag/AgCl

1- Barakat, N.A.M.; Ali Abdelkareem, M.; Abdelghani, E.A.M. Influence of Sn Content, Nanostructural Morphology, and Synthesis Temperature on the Electrochemical Active Area of Ni-Sn/C Nanocomposite: Verification of Methanol and Urea Electrooxidation. *Catalysts 9* (2019) 330. https://doi.org/10.3390/catal9040330

2- Gracita M. Tomboc, Medhen W. Abebe, Anteneh F. Baye, Hern Kim, Utilization of the superior properties of highly mesoporous PVP modified NiCo2O4 with accessible 3D nanostructure and flower-like morphology towards electrochemical methanol oxidation reaction, Journal of Energy Chemistry 29 (2019) 136-146. https://doi.org/10.1016/j.jechem.2018.08.009

3- Dilan Atbas, Aykut Çağlar, Hilal Kivrak, and Arif Kivrak, "Microwave Assisted Synthesis of Sn Promoted Pt Catalysts and Their Ethanol Electro-oxidation Activities." American Journal of Nanomaterials 4 (1),(2016): 8-11. doi: 10.12691/ajn-4-1-2.

4- Barakat NAM, Amen MT, Al-Mubaddel FS, Karim MR, Alrashed M. NiSn nanoparticle-incorporated carbon nanofibers as efficient electrocatalysts for urea oxidation and working anodes in direct urea fuel cells. J Adv Res. 16(16) (2018) 43-53. doi: 10.1016/j.jare.2018.12.003.

5- Amal Zaher, Waleed M.A. El Rouby, Nasser A.M. Barakat, Tungsten incorporation in nickel doped carbon nanofibers as efficient electrocatalyst for ethanol oxidation, Fuel 280 (2020) 118654. https://doi.org/10.1016/j.fuel.2020.118654
